# Introducing a Short Measure of Shared Servant Leadership Impacting Team Performance through Team Behavioral Integration

**DOI:** 10.3389/fpsyg.2015.02002

**Published:** 2016-01-07

**Authors:** Milton Sousa, Dirk Van Dierendonck

**Affiliations:** ^1^Leadership Knowledge Centre, Nova School of Business and EconomicsLisbon, Portugal; ^2^Centre for Leadership Studies, Rotterdam School of Management, Erasmus UniversityRotterdam, Netherlands

**Keywords:** servant leadership, shared leadership, team behavioral integration, self-managed teams, measure

## Abstract

The research reported in this paper was designed to study the influence of shared servant leadership on team performance through the mediating effect of team behavioral integration, while validating a new short measure of shared servant leadership. A round-robin approach was used to collect data in two similar studies. Study 1 included 244 undergraduate students in 61 teams following an intense HRM business simulation of 2 weeks. The following year, study 2 included 288 students in 72 teams involved in the same simulation. The most important findings were that (1) shared servant leadership was a strong determinant of team behavioral integration, (2) information exchange worked as the main mediating process between shared servant leadership and team performance, and (3) the essence of servant leadership can be captured on the key dimensions of empowerment, humility, stewardship and accountability, allowing for a new promising shortened four-dimensional measure of shared servant leadership.

## Introduction

**Figure 1:**
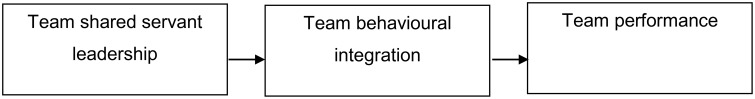
**Conceptual model relating the variables of shared servant leadership, team behavioral integration, and team performance**.

This article explores the role of shared servant leadership in affecting team performance through the mediating role of team behavioral integration. We bring forward the idea that in a self-managed team, the collective composition of servant like leadership behaviors by individual team members can be conducive of team performance. This is based on the principle that leadership can be seen as a process emerging from the interaction between agents, as opposed to an influencing process that flows from a central leader alone. Such a collectivist view is reflected in models such as team, shared, complex, network and collective leadership (Yammarino et al., 2012). We hypothesize that, when combined, the servant leadership behaviors advanced by Van Dierendonck and Nuijten (2011), based on empowerment, stewardship, humility and accountability, will be particularly applicable in the context of shared leadership, as they naturally reflect and support a process whereby “it is only the collective that matters and single leaders *disappear* so to speak” (Yammarino et al., 2012; p. 398). We further advance that improved team behavioral integration, in its three aspects of joint decision making, information exchange and collective behavior, will be the mechanism through which shared servant leadership will have an impact on the team's performance. Figure 1 depicts the conceptual model that guides this research.

In addition to this conceptual proposition, through our studies, we introduce a short measure of servant leadership, based on the original instrument by Van Dierendonck and Nuijten's (2011). Traditional team rating surveys are relatively simple (each member rates the overall team leadership) but rather inaccurate in assessing shared leadership (e.g., Pearce and Sims, 2002; Avolio et al., 2003). Round-robin approaches, whereby each team member assesses everyone else in the team individually from which a team level score is composed, provide much more accuracy but are time-consuming, especially when surveys are long. Having a shorter survey that reduces completion time while keeping the essence of the original measure would be useful and practical. This new measure will be a more practical tool for assessing shared leadership through a collective assessment of the leadership present in the team (Gockel and Werth, 2010), with the improved accuracy of a round-robin data collection method.

In order to test our conceptual model and validate the new short measure, two studies were conducted based on confirmatory factor analysis. We start by further elaborating on the concepts and corresponding constructs of shared leadership, servant leadership and team behavioral integration, as well as the underlying principles supporting the relationship between them.

## Constructs and model operationalization

### Shared leadership

Shared leadership is defined as “a dynamic, interactive influence process among individuals in groups for which the objective is to lead one another to the achievement of group or organizational goals or both” (Pearce and Conger, 2003; p. 1). Shared leadership changes the focus from a vertical leadership approach where one leader influences several followers to a horizontal approach where leadership becomes a joint activity of the team members showing leadership behavior toward each other (Bligh et al., 2006). Research on shared leadership has already shown its potential use in better understanding team effectiveness in terms of ratings by managers, customers and self-ratings (e.g., Pearce and Sims, 2002; Hoch et al., 2010).

Shared leadership gains increased relevance in the context of self-managed teams, as the absence of a clear hierarchy likely provides fertile ground for shared leadership to emerge. The ideas behind self-managed teams originate from socio-technical systems theory (Stewart and Manz, 1995). It is a way of organizing that combines both the social and the technical aspects of work. Instead of working as individuals with individual targets, employees work together in teams and are jointly responsible for team targets. With the absence of a direct supervisor, these teams have relatively more freedom to plan their own work. This can bring a strong sense of empowerment within the individual team members and opens the way for a more shared form of leadership instead of the more traditional hierarchical types. One needs to bear in mind that despite the absence of an appointed leader within a self-managed team, some kind of informal leadership will likely appear (Wolff et al., 2002). Team leaders help define team objectives, keep a team focused on team goals, and provide coordination between team members. Even in self-managed teams these roles are necessary. What distinguishes shared leadership from centralized leadership, especially in self-managed teams, is that these leadership roles are often fulfilled by different team members instead of only one, in a fluid process. As was proposed by West et al. (2003), a lack of leadership clarity can be detrimental for team performance, especially if this leads to conflict over the leadership role or the direction that a team should take. However, when one sees leadership as a process instead of a single one-to-many power relationship, this clarity can be achieved through a mutually reinforcing shared leadership process.

### The operationalization of shared leadership

Capturing shared leadership in teams is not easy. Previous attempts have often focused on the influence of the team as a whole or on how team members in general show leadership behavior (Gockel and Werth, 2010). For example, Pearce and Sims (2002) asked participants to rate their team members jointly on shared leadership. A similar approach was used by Avolio et al. (2003) and in a more recent study by Hoch et al. (2010). Basically, items from leadership measures are reformulated from “my leader…” into “my team members….” The main disadvantage of these measures is their lack of accuracy as one cannot know the point of reference taken by respondents when evaluating the team as a whole (Gockel and Werth, 2010). In order to overcome this problem, in the present study, shared leadership is measured through a round-robin approach whereby team members are individually assessed on their servant leadership behaviors toward each respondent, which makes it possible to consider it a relational construct (Mayo et al., 2003). The collective team average is then calculated, representing the total amount of servant leadership behavior demonstrated in the team, which should be more accurate than asking participants to rate the team as a whole. While this method has similarities with the social network analysis methods suggested by Gockel and Werth (2010) in terms of data collection, it has some distinct differences with regard to interpretation. In social network analysis, shared leadership is assessed mainly through the measures of centralization and density (Gockel and Werth, 2010). Such measures are indirect characteristics of the network topography, as they provide ratios instead of actual leadership scores. However, as we aimed to validate the short measure of servant leadership, we needed to ensure a direct measure of the amount of shared servant leadership in the team instead of using indirect ratios. We see therefore our approach as an extension and improvement of the team rating approach suggested by Gockel and Werth (2010) through the inclusion of round-robin measures of servant leadership, helping to overcome the inaccuracy of team level measures.

### Servant leadership as a model for shared leadership

Robert Greenleaf (1904–1990) introduced the notion of servant leadership after reading Herman Hesse's Journey to the East (Greenleaf, 1977). This book portrays the archetype of a servant-first leader that inspired Greenleaf to extrapolate this notion to the context of modern organizations. Greenleaf's (1977) concept of servant leadership is very much focused on this initial motivation to serve as the following quote testifies: “The servant-leader is servant first…It begins with the natural feeling that one wants to serve, to serve first. Then conscious choice brings one to aspire to lead” (Greenleaf, 2002; p. 7). As such, the servant leader's major concern is the development and growth of others. Spears (1996; p. 33) highlights how “servant leadership emphasizes increased service to others; a holistic approach to work; promoting a sense of community; and the sharing of power in decision making.”

The relevance of servant leadership for team functioning has been demonstrated in several recent studies that focused on servant leadership in a hierarchical setting. Walumbwa et al. (2010) showed that team level servant leadership was related to higher individual organizational commitment, self-efficacy and supervisor rated organizational citizenship behavior. Hu and Liden (2011) found that team-level servant leadership was related to team performance, team organizational citizenship behavior and team potency. The results of Schaubroeck et al. (2011) are similar in that they compared team-level transformational leadership with team-level servant leadership and showed that servant leadership was related to team performance through affect-based trust in the leader and team psychological safety. All three studies confirm the relevance for team functioning of servant leadership as shown by the direct supervisor. The present study builds on their insights by its focus on shared servant leadership in self-managed teams without a direct supervisor. We advance that aspects of servant leadership such as a servant-first attitude (Greenleaf, 1977), humility (Russell, 2001; Patterson, 2003; Van Dierendonck, 2011), and the ability to perform while focusing on the good of the whole (Van Dierendonck, 2011) will be supportive of the antecedents of shared leadership suggested by Carson et al. (2007) such as shared purpose, social support or having a voice.

Given its increasing adoption and validity in different cultural settings including the Netherlands, UK, Italy, Finland and Portugal (Hakanen and Van Dierendonck, 2011; Van Dierendonck and Nuijten, 2011; Bobbio et al., 2012; Sousa and van Dierendonck, 2014), we opted to use the measurement development study by Van Dierendonck and Nuijten (2011) providing a rather comprehensive and solid instrument, based on 8 dimensions and 30 items. These include: empowerment (7 items), accountability (3 items), standing back (3 items), humility (5 items), authenticity (4 items), courage (2 items), forgiveness (3 items), and stewardship (3 items). From this whole set, the dimensions of empowerment, stewardship, accountability and humility were suggested by Van Dierendonck and Nuijten (2011) as forming core aspects of servant leadership behavior. With this study we aim to confirm these four dimensions as the essential attributes of shared servant leadership within a self-management team context. Given the extensive amount of mutual one-to-one estimates between team members to calculate shared servant leadership in a round-robin approach, such a shortened measure will also prove far more practical. We posit that team members who show servant leadership behavior will actively empower and develop other team members, show humility toward one another, provide direction in day-to-day work by mutually holding others accountable, and emphasize the importance to act as stewards who work for the good of the team as a whole. In the next chapter we further elaborate on the specific impact of these dimensions of servant leadership, as a shared process, on team behavioral integration.

### Shared servant leadership and team behavioral integration

Team behavioral integration was suggested to be a key fundamental aspect of collective leadership (Friedrich et al., 2009; Yammarino et al., 2010, 2012). Likewise, we posit that shared servant leadership will be reflected in higher levels of team behavioral integration. As such, team behavioral integration is introduced into our theoretical model as a mediating variable to help understand the possible beneficial influence of shared leadership on team performance.

Hambrick (1994; p. 188) defined team behavioral integration as “the degree to which the group engages in mutual and collaborative interaction.” Originally proposed to capture effective functioning performance in top management teams, team behavioral integration consists of three interrelated components that capture both social and task related dimensions (Hambrick, 1994; Siegel and Hambrick, 1996). The social dimension is captured on the component of collaborative behavior (Hambrick, 1994), which builds on the concept social integration. Social integration is mainly an affective construct reflected on social interaction, attraction to the group and satisfaction with other team members (O'Reilly et al., 1989). This affective and emotional bond carries however risks on the quality of decision making due to the fear of damaging relationships within the team. Consequently, for social integration to be effective it needs therefore to be combined with other task related behaviors (Schweiger and Sandberg, 1989). Hambrick (1994) encapsulated these task related behaviors in the constructs of joint decision making and information exchange.

Simsek et al. (2005) further elaborate on these three different dimensions. The authors support that collective behavior can be translated into behaviors of mutual support in managing workload, flexibility about switching responsibilities to make work easier for each other and the willingness of team members to help each other in meeting deadlines (Simsek et al., 2005). Joint decision making can be observed through the care in communicating interdependencies among team members, creating shared understanding of joint problems and each other's needs, as well as openly discussing expectations of each other (Simsek et al., 2005). Concerning information exchange, Simsek et al. (2005) consider both quantitative (e.g., number of ideas being created) and qualitative aspects (e.g., level of creativity and innovation, and the quality of proposed solutions toward problems).

The relevance of team behavioral integration for team performance was particularly emphasized by three studies that related top management team behavioral integration to company performance (Simsek et al., 2005; Lubatkin et al., 2006; Carmeli, 2008). Other studies showed its relevance for individual improvisation (Magni et al., 2009) and better quality of strategic decisions (Carmeli and Schaubroeck, 2006). On a related note, team leadership has been positioned as essential for developing shared mental models, collective information processing and team metacognition (Zaccaro et al., 2001). Also, shared leadership in teams has been related to greater collaboration, coordination, cooperation and group cohesion (Ensley et al., 2003), which are similar concepts to the three dimensions of team behavioral integration.

Given these considerations, as a people-centered mutually supporting leadership model, we suggest that the aggregate composition of servant leadership behaviors by individuals toward one another (shared servant leadership), will likely directly or indirectly enhance team behavioral integration. In the following text, we note some particularly noteworthy potential linkages based on the four dimensions of servant leadership outlined before as essential for the shared leadership context.

*Empowerment* refers to a motivational concept which includes empowering leadership behavior for encouraging self-directed decision making, information sharing, and coaching for innovative performance (Konczak et al., 2000). Based on this definition empowerment seems to affect both information exchange and joint decision making, as it opens up the channels of communication in support of joint coordination. Empowerment is also a base condition for shared leadership to emerge (Yammarino et al., 2012), whereby team members are able to trust each other on their ability to perform different tasks. It means that team members mutually encourage taking initiative, diligently share information, support each other in decision making and help others understanding new challenges and topics. In teams demonstrating high levels of shared leadership, one would expect members to often agree on sharing tasks such that those less knowledgeable can grow and learn, in a true mutually empowering fashion. Within team behavioral integration, this mutually supporting orientation can be captured in the social dimension of collective behavior.

*Humility* is about modesty reflected in a servant-leader's tendency to give priority to the interest of others, acknowledging mistakes and giving room to learn. For shared leadership to emerge in a team it is essential that the team members are able to acknowledge their limitations and the fact that other people can contribute in different ways and according to their level of development. In addition, as a collectivist form of leadership, shared leadership means that any individual team member needs to be able to move into the background when necessary (Yammarino et al., 2012). This allows others to assume leading roles as demanded by the task at hand. From a task oriented point of view, humility will support the quantity of information exchange by acknowledging the value of everyone's contribution and ideas. At the same time, humility can affect joint decision making as it will instill a culture of dialog and genuine interest in mutual understanding through humble inquiry (Schein, 2013), fostering creativity and innovative thinking. From a social integration perspective, humility can support collective behavior as it amplifies the importance of the whole above self-interest and creates a space for reaching out to other team members when in need.

*Accountability* is about providing direction taking into account other people's abilities, needs, and input, while holding them responsible for their achievements. In a team with shared leadership this role might be partaken among several members or eventually rotated. It also means that all members assume responsibility for each other's work and will mutually hold each other accountable for their contribution. This shared responsibility and accountability forms a cornerstone of shared leadership behavior (Pearce and Conger, 2003). In addition, accountability is mostly associated with the practical aspects of work, also present in servant leadership. Defining tasks, work processes, objectives, deadlines and control mechanisms remains critical for work to be done effectively. As such, this dimension will likely be most relevant for the task related aspects of team behavioral integration. It can support joint decision making as it emphasizes the need to mutually agree on targets, task assignments, methods and processes while ensuring execution and performance. At the same time, accountability can stimulate both the quantity and the quality of information exchange. On the quantity of information, it can prevent team members from free riding or not being sufficiently involved, increasing the number of ideas and solutions being considered. On the quality aspect, accountability will make members thoughtful of the relevance and effectiveness of their ideas and proposed solutions.

*Stewardship* refers to stimulating others to act in the common interest and to take a viewpoint that focuses on the good of the whole. These core aspects have been shown to contribute to followers experiencing a more challenging work setting, a sense of psychological empowerment and higher organizational commitment (Asag-gau and Van Dierendonck, 2011). This aspect of servant leadership brings an element of self-transcendence, by putting others and the mission above the self. In light of this definition, when all team members act as stewards, it becomes accepted that the team is more important than any individual, again a base condition for shared leadership to emerge (Pearce and Conger, 2003; Yammarino et al., 2012). From an affective perspective, stewardship seems therefore to be particularly relevant for the aspect of collective behavior, as it emphasizes the importance of the whole and staying on course to achieve the team's objectives. From a task oriented point of view, stewardship will enable joint decision making as it stimulates team members to understand joint problems and each other's needs in the context of a larger picture. It can also support information exchange by increasing the quality of solutions being proposed through a higher focus on the relevant team challenges that need to be addressed (ensuring the relevance of information being exchanged).

In light of the possible links established above, it can be expected that if team members on average show more of these mutual and supportive servant leadership behaviors toward each other (empowerment, humility, accountability, and stewardship), team behavioral integration will be strengthened, which will lead to better overall team performance. Together the above reasoning can be summarized in the following hypotheses:

1. Hypothesis 1: Given its emphasis on mutual empowerment, accountability, stewardship and humility, shared servant leadership will be positively related to team behavioral integration.
2. Hypothesis 2: As a reflection of good team functioning, team behavioral integration will operate as a mediating variable between shared servant leadership and team performance.

In a nutshell, the present study aims to test the mediating effect of shared servant leadership on team performance through team behavioral integration. A round-robin approach was used to collect the data, which allows for a more accurate measure of the amount of shared servant leadership in a team. The study also aims to validate a short measure of servant leadership for the shared leadership context based on the four key dimensions of empowerment, stewardship, accountability and humility. Several control variables were included to take into account possible third variable effects, namely academic competence and team familiarity.

## Methods study 1

### Data collection

In order to validate our model, it was important to have a large sample of self-managed teams (without a formal leader) going through a similar assignment and in comparable contextual circumstances, to allow for objective performance comparison. This is hard to find in an organizational context. For that reason, a simulation based assignment in a Business Administration program was used.

The simulation is based on realistic scenarios extracted from real cases pertaining to the activities of Human Resource Management (HRM) departments. The simulation lasts 2 weeks with intense teamwork in groups of four. It is important to note that during this 2 week period this assignment is their only activity for the course, reducing interference, and conflicting goals. The simulation is a major activity requiring substantial time and focus. Each team represented the HRM department of a company where HR relevant decisions had to be made for the company. These decisions, concerning realistic recruitment, selection and retention policies, had to be taken on a daily basis for 8 days. In the morning, feedback was given on how their company was doing in comparison to the companies of the other teams. New decisions had to be taken before the end of each day. It is important to note that no leader was appointed in the teams. They were instructed to function as a self-managed team. The participants were asked to fill out a survey on their team functioning, 1 week after the simulation directly following handing in their final report, giving extra course credits. Data has been collected through an online survey in accordance with the ethical guidelines of the American Psychological Association. The ethical rules and regulations of the University were also applied. As such, (i) participation was completely voluntary, (ii) data collection through a self-report survey is exempted from an institutional ethics committee's approval, and (iii) the subjects filled out the survey for extra course credit (no money has been given). Subjects were informed about the nature of the study on the first webpage. Informed consent was given by clicking on the “Next” button.

#### Participants

Respondents were third year undergraduate Business Administration students participating in a HRM course. Only the results of the teams that had all four members filling out the surveys were included in the study. This provides a full database with reports of all team members on each other. The sample included 61 teams, totalling 244 students (response percentage of 71%). Of them 65 % were male and 35% female. The average age was 21.0 (*SD* = 1.5) years.

### Measures

#### Shared servant leadership

All participants were asked to rate the leadership behavior they perceived from their fellow team members in a round-robin fashion (whereby every team member evaluates all other team members' behaviors). For the developmental purpose of this survey, where we also wanted to test the validity of the short measure, all 30 items from the Servant Leadership Survey (SLS; Van Dierendonck and Nuijten, 2011) were incorporated. As explained before, items were reformulated to indicate the level of servant leadership shown by each team members toward the person filling out the survey (i.e., instead of asking questions in relation to the leader, they were asked in relation to each team member individually). In addition, questions were provided in the past tense, referring to the period of the project, as opposed to a stable management relationship from the original survey. For example, one of the original empowerment items was reformulated from “My manager gives me the information I need to my work well” to “< name of team member> gave me the information I needed to do my work well.” In another example, this time from the accountability dimension, the following item was rephrased from “My manager holds me responsible for the work I carry out” to “< name of team member> held me responsible for the work I carried out.” Such adaptations were done for all 30 items from the original survey. Ratings were to be given on a 5-point Likert scale, ranging from *never* to *very often*. For all participants, the answers to all items were averaged to indicate the mean level of the servant leadership behavior as received from the other team members.

Based on this data, team shared servant leadership becomes the combined servant leader behavior of team members shown toward one another. This gives an indication of the average level of shared leadership in a team, which is similar to approach 1 in the Gockel and Werth (2010) paper but with the advantage of including round-robin measures for a more accurate assessment of the total average amount of shared leadership. One should note that there is no need for checking for consensus among the different team members because items refer to the servant leadership behavior shown by each team member individually and not on the overall servant leadership level of the team.

#### Team behavioral integration

Team behavioral integration was measured with the three-dimensional measure developed by Simsek et al. (2005), including collective behavior, information exchange and joint decision making. Each dimension was measured with three items. Before aggregating the data to team level, the consensus among the different team members was checked with regard to their assessment of team behavioral integration. The Rwg(j) scores (James et al., 1984) were calculated. As an additional test, the intraclass correlation (ICC1) was also calculated. This correlation gives an estimate of the related consistency among the team members. We also tested whether the operationalization of team behavioral integration acknowledged its three-dimensional conceptualization.

#### Team performance

During the simulation, the teams received feedback about their performance on several company indicators, generated by the simulation software. These indicators were also transformed into an overall score which was communicated to the teams after each round. Performance in this paper is their final ranking on the simulation, which gives an indication of their overall performance throughout the eight decision rounds. Their overall end score was differentiated between 6 (for the teams whose score belonged to the lowest 10%) and 10 (for groups belonging to the highest 10%).

#### Control variables

Past research has argued that team member familiarity may affect team performance (e.g., Gruenfeld et al., 1996). Therefore, we took in member familiarity as a control variable. Respondents were to judge how well they knew each team member on a scale from 1 (*not at all*) to 5 (*very well*). These scores were added together and aggregated to team level to create a team score of familiarity. Academic competence of the individual team members may also influence team performance. Respondents were asked to give an estimate of their average grade of other courses. Course grade is used as a proxy for general mental capacity, their learning style, and their motivation to put in an effort to reach high grades. These individual scores were averaged within a team for a score of a team's average academic competence. No index for within group agreement was calculated for the control variables as team members are not necessarily similar in the degree to which they know their fellow team members, nor in their average grade. In this situation the team average would still be an accurate reflection of member familiarity and intellectual capacity (cf. Gruenfeld et al., 1996).

### Assessing the validity of the new servant leadership short measure

The factorial validity of the hypothesized four-dimensional structure (humility, empowerment, stewardship, and accountability) of the new shortened version of the servant leadership survey was tested in both studies. The mean item-scores across team members were used as input for Mplus 6 (Muthén and Muthén, 2009). The nested (multi-level) structure of the dataset (i.e., participants in teams) was accounted for, thereby guaranteeing the correct error variances. In addition, in order to test the validity of the shortened version, in study 1 we compared the underlying variance of the full servant leadership scale with all 30 items with 8 dimensions to that of the reduced version with 15 items and only 4 dimensions. A model was tested where the four dimensions were allowed to load together on one second-order factor. In addition, all 30 items of the original scales were allowed to load on one underlying factor. This factor signifies the total underlying servant leadership variance of the full measure. The second order servant leadership factor (representing the underlying variance of the four dimensions theorized to be most important for shared servant leadership in self-managed teams) was allowed to correlate with the leadership factor which was determined by all 30 items.

### Model validation

Following Anderson and Gerbing (1988), we first tested the adequacy of the measurement model of the latent constructs using Mplus 6 (Muthén and Muthén, 2009) before actually testing the relations in the full model. To operationalize the latent construct of servant leadership, the four dimensions were used as manifest indicators. For the three team behavioral integration sub-dimensions, the items of each scale were used as indicators. In this way these latent constructs were determined by three or four indicators, which is the recommended practice if the goal is to study a variable at an overall level of generality and one wants to reduce the level of nuisance and bias that may come from working with the separate items directly (Bandalos, 2002). Team performance, academic competence and team familiarity were used as manifest variables. After validating the measurement model, the conceptual model was tested in both studies, with structural equation models with latent and manifest variables using Mplus 6 (Muthén and Muthén, 2009). As a final confirmation step, the indirect effects of the most significant mediating factors of team behavioral integration were tested with bootstrapping (Preacher et al., 2008).

## Methods study 2

The setup of the second study is in essence a replica of the first one but this time based on students from the following year. This allowed again for a more accurate comparison between results from both studies. The aim of the second study was to confirm the findings of the first study. As such, during study 2, only the short measure of servant leadership validated in study 1 was used (15 items) with all other measures being exactly the same.

Like in the first study, participants were third year undergraduate Business Administration students participating in a HRM course that included a HRM-simulation of 2 weeks with intense teamwork in groups of four. Only the results of the teams that had all four members filling out the surveys were included in the study. This provides a full database with reports of all team members on each other. The sample included 72 teams, totalling 288 students (response percentage of 72%). Of them 62% were male and 38% female. The average age was 20.9 (*SD* = 1.3) years. The same ethical rules and regulations of for data collection from the first study were applied to this second study.

## Results study 1

### Validity of the short shared servant leadership measure

Before testing the whole model, the validity of the short measure was evaluated. Initially, the fit of the hypothesized 4-dimensional structure corresponding to the short version of the servant leadership measure was compared to a 1-dimensional structure (all items loading on one leadership dimension). The fit indices were *X*^2^ = 263, 887, *df* = 129, CFI = 0.91, TLI = 0.89, RMSEA = 0.07, SRMR = 0.07, for the 4-dimensional model, and *X*^2^ = 648.989, *df* = 135, CFI = 0.66, TLI = 0.61, RMSEA = 0.13, SRMR = 0.10, for the 1-dimensional model. The 4-dimensional model clearly shows the best fit, confirming the underlying multi-dimensional structure of servant leadership within this context. However, one comparative fit index (TLI) was still below 0.90, indicating some misfit in the measurement model. Items that either loaded low (i.e., a standardized factor loading lower than 0.40) on their proposed dimension or where the modification indices indicated a cross-loading on one of the other dimensions were removed. This resulted in the removal of three items from empowerment, humility and stewardship (one item from each subscale). The resulting 4-dimensional model had excellent fit indices (*X*^2^ = 139.185, *df* = 84, CFI = 0.93, TLI = 0.92, RMSEA = 0.06, SRMR = 0.06). The 4-dimensional model with one underlying dimension showed a comparable fit: *X*^2^ = 157.561, *df* = 86, CFI = 0.94, TLI = 0.93, RMSEA = 0.06, SRMR = 0.06. These results confirm that the short shared servant leadership measure in this paper is a 4-dimensional concept with one underlying second order factor. The internal consistencies are 0.80 for empowerment (6 items), 0.88 for accountability (3 items), 0.60 for stewardship (2 items), and 0.75 for humility (4 items). Overall, the reliability of these subscales is good. Please note that internal consistency also depends on the number of items. Stewardship has only two items, 0.60 is with only two items still respectable (as will be seen later, this value is higher in study 2).

As explained before, in order to test the validity of the shortened version, we compared the underlying variance of the full servant leadership scale with all 30 items with 8 dimensions to that of the reduced version with 15 items and only 4 dimensions. The correlation between the factor capturing the full range of the servant leadership measure and the one representing the shortened measure was 0.90. In other words, the short scale consisting of only 4 out the 8 dimensions and half the number of items (15 instead of 30), still represents 81% of the variance of the full scale.

### Validity of the team behavioral integration construct

Concerning the mediating variable, team behavioral integration, it was important to assess its three-dimensional nature. Indeed, the three-dimensional model, showed a much better fit compared to the one-dimensional model (*X*^2^ = 42.24, *df* = 24, CFI = 0.93, TLI = 0.90, RMSEA = 0.11, SRMR = 0.07, vs. *X*^2^ = 121.49, *df* = 27, CFI = 0.64, TLI = 0.51, RMSEA = 0.24, SRMR = 0.13). The internal consistencies were 0.85 for collective behavior (3 items), 0.75 for information exchange (3 items), and 0.74 for joint decision making (3 items). In addition, as a collective assessment, we wanted to confirm the extent to which behavioral integration results could be aggregated at team level. The Rwg(j) scores were 0.86 for collective behavior, 0.92 for information exchange and 0.78 for joint decision making. Additional insight is gained through the intraclass correlation (ICC1). The ICC1 scores were 0.19 for collective behavior, 0.16 for information exchange and 0.34 for joint decision making. Overall, it can be concluded that there is enough overlap between team members to calculate average team behavioral integration scores.

### Validity of the measurement model

The output variable for team performance and the control variables of academic competence and team familiarity were used as manifest variables. Following Anderson and Gerbing (1988), we tested the adequacy of the measurement model of the latent constructs of shared servant leadership and team behavioral integration before actually testing the relations in the full model. The relative fit indices were excellent (*X*^2^ = 73, 569, *df* = 59, CFI = 0.96, TLI = 0.95, RMSEA = 0.06, SRMR = 0.07), confirming our operationalization of shared servant leadership and team behavioral integration with four and three separate constructs, respectively.

### Validity of the conceptual model

Once the measurement model was confirmed, the hypothesized model was tested, showing only a moderate fit (*X*^2^ = 146.286, *df* = 95, CFI = 0.88, TLI = 0.84, RMSEA = 0.09, SRMR = 0.09). By checking the significance of the paths and the modification indices, several improvements were suggested. Interestingly, neither control variable (average team academic competence or team familiarity) were significantly related to team performance. As a result, they were removed from the model. Additionally, the paths between collective behavior and joint decision making and team performance were not significant. The adjusted model with the non-significant paths fixed at zero has an excellent fit. (*X*^2^ = 91.645, *df* = 71, CFI = 0.95, TLI = 0.93, RMSEA = 0.07, SRMR = 0.08). Table 1 shows the individual mean values, standard deviations and intercorrelations of the variables of study 1.

**Table 1:** **Descriptives and intercorrelations of study variables at team level—study 1**.

	***M***	***SD***	**1**	**2**	**3**	**4**	**5**	**6**	**7**	**8**	**9**
1. Academic competence	70.14	2.87									
2. Team familiarity	3.46	0.83	0.15								
3. Collective behavior	6.14	0.48	0.13	−0.15							
4. Information exchange	4.94	0.47	−0.01	−0.10	0.38^*^						
5. Joint decision making	5.31	0.41	−0.01	0.15	0.53^*^	0.43^*^					
6. Empowerment	3.36	0.34	0.00	0.28^*^	0.38^*^	0.39^*^	0.57^*^				
7. Accountability	3.41	0.43	0.18	0.37	−0.00	0.08	0.33^*^	0.40^*^			
8. Stewardship	3.91	0.40	0.29^*^	−0.02	0.20	0.08	0.32^*^	0.51^*^	0.11		
9. Humility	3.01	0.90	0.13	0.34	0.25^*^	0.31^*^	0.37^*^	0.73^*^	0.31^*^	0.48	
10. Team performance	7.92	1.34	−0.08	−0.12	0.10	0.56^*^	0.16	0.08	−0.16	−0.11	0.12

*n = 61*.

* *p < 0.05*.

**Figure 2:**
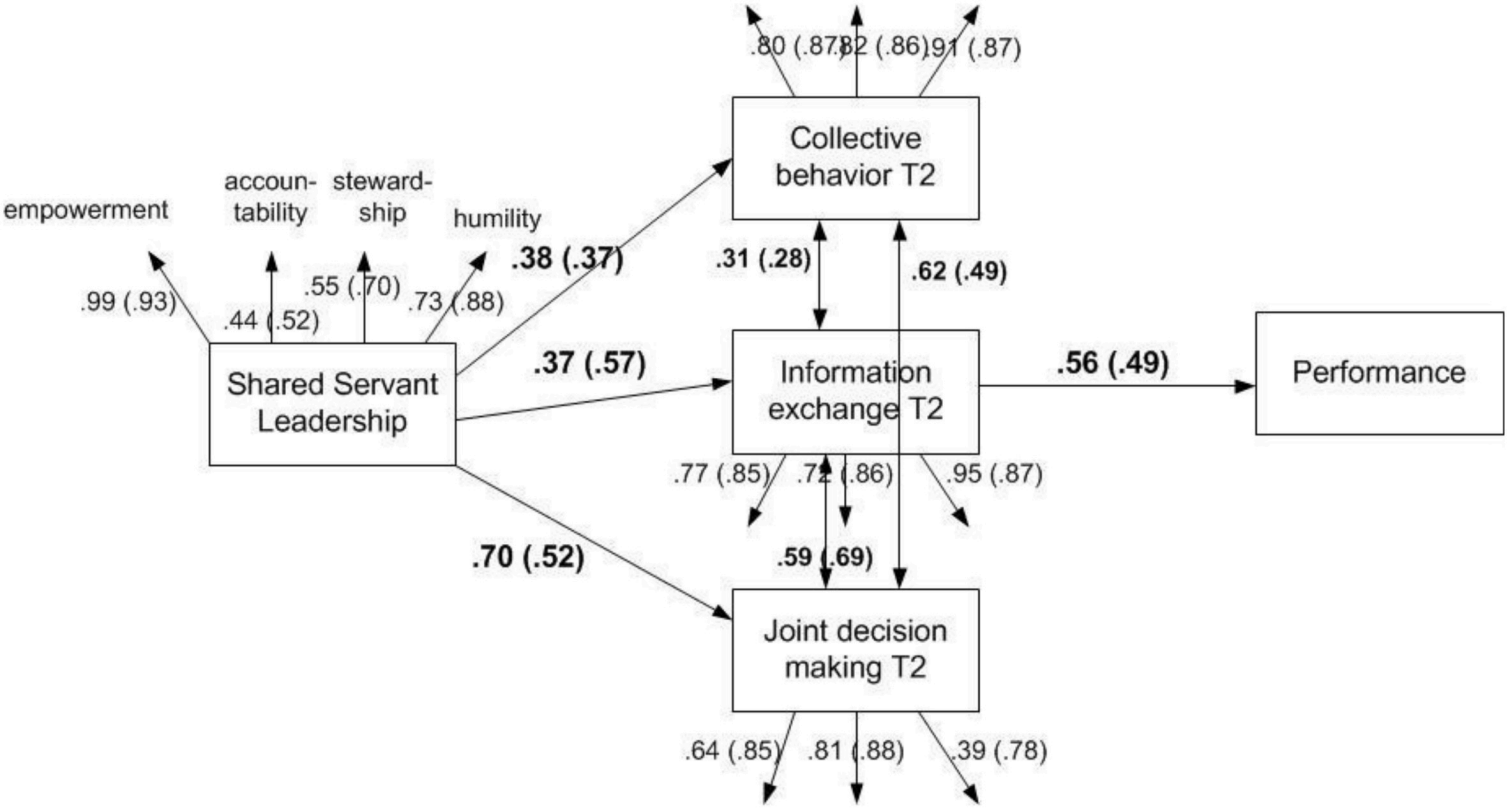
**Shared servant leadership, team behavioral integration, and team performance, Empirical model for study 1 and study 2**. Depicted are the standardized values. Between brackets are the values for study 2.

Figure 2 shows the standardized model. As it can be seen, shared servant leadership is related to all three elements of behavioral integration. This shows that shared servant leadership behavior within self-managed teams is closely related to a stronger collective functioning. There is also an indirect relation to performance, notably through information exchange in the team.

In a final step, this indirect role of information exchange in the relation between servant leadership and team performance was tested with bootstrapping (Preacher et al., 2008). The standardized estimated indirect coefficient was 0.21 (*p* = 0.01; 95% confidence interval ranged between 0.07 and 0.34), confirming its mediating role.

### Conclusion

This first study seems to confirm the first hypothesis that shared team servant leadership does have an effect on team behavioral integration. Concerning the second hypothesis, within the team behavioral integration construct, information exchange seems to play a more prominent role as a mediating variable, which confirms the prominence of this factor as suggested by Yammarino et al. (2012). The fact that academic competence and team familiarity do not seem to influence this set of relations only comes to strengthen the apparent power of shared servant leadership on bringing teams to a higher performing level.

Another important and promising development from this first study is the validity and reliability of the short measure for shared servant leadership based on 4 dimensions and 15 items, as opposed to the original consisting of 8 dimensions and 30 items. This allows capturing the essence of shared servant leadership as a model based on four key dimensions: humility, empowerment, stewardship and accountability as advanced in our third hypothesis. On a more practical level, this short measure eases research through the reduced number of items in the survey, which is quite relevant when using an extensive round-robin approach to measure team shared leadership.

In order to confirm the conclusions and findings explained before, a second similar study was developed. The findings of this second study will now be explained.

## Results study 2

### Validity of the short shared servant leadership measure

The fit of the new developed 4-dimensional measure from was compared to a 1-dimensional structure (all items loading on one leadership dimension). The fit indices were *X*^2^ = 165.896, *df* = 84, CFI = 0.95, TLI = 0.94, RMSEA = 0.06, SRMR = 0.05, for the 4-dimensional model, and *X*^2^ = 535.909, *df* = 90, CFI = 0.73, TLI = 0.68, RMSEA = 0.13, SRMR = 0.09, for the 1-dimensional model. The 4-dimensional model with one underlying dimension showed a comparable fit: *X*^2^ = 165.149, *df* = 86, CFI = 0.95, TLI = 0.94, RMSEA = 0.06, SRMR = 0.05. The standardized factor loading of the sub-dimensions on the second order factor were: 0.94 for empowerment, 0.51 for accountability, 0.88 for stewardship, and 0.96 for humility. The internal consistencies were 0.81 for empowerment (6 items), 0.90 for accountability (3 items), 0.69 for stewardship (2 items), and 0.77 for humility (4 items). Taken together, these results confirm the factorial validity of the shared servant leadership measure as developed in study 1, as a 4-dimensional concept with one underlying second order factor.

### Validity of the team behavioral integration construct

The internal consistencies for the team behavioral integration measure were 0.89 for collective behavior (3 items), 0.86 for information exchange (3 items), and 0.90 for joint decision making (3 items). All demonstrating good results. As in study 1, we checked the overlap between team members in their estimation to confirm our use of aggregated team scores for team behavioral integration. The Rwg(j) scores (James et al., 1984) were 0.91 for collective behavior, 0.94 for information exchange and 0.89 for joint decision making. The ICC1 scores were 0.31 for collective behavior, 0.59 for information exchange and 0.41 for joint decision making, again allowing us to aggregate results at team level.

### Validity of the conceptual model

Next, the model from study 1 was tested in this study to see if it could be replicated with an independent sample within a similar setting. The latent model was determined in the same way as in study 1. The fit was again good: *X*^2^ = 112.966, *df* = 71, CFI = 0.94, TLI = 0.92, RMSEA = 0.09, SRMR = 0.07. There were no significant improvements suggested by the modification indices. The indirect role of information exchange in the relation between servant leadership and team performance was again tested with bootstrapping (Preacher et al., 2008). The standardized estimated indirect coefficient was 0.28 (*p* < 0.001; 95% confidence interval ranged between 0.16 and 0.40), confirming its mediating role. The standardized factor loadings of the resulting model can be found between brackets in Figure 2. Table 2 shows the individual mean values, standard deviations and intercorrelations of the variables of study 2.

**Table 2:** **Descriptives and intercorrelations of study variables at team level—study 2**.

	***M***	***SD***	**1**	**2**	**3**	**4**	**5**	**6**	**7**
1. Collective behavior	6.00	0.42							
2. Information exchange	5.17	0.49	0.38^*^						
3. Joint decision making	5.35	0.50	0.47^*^	0.70^*^					
4. Empowerment	3.37	0.38	0.31^*^	0.47^*^	0.46^*^				
5. Accountability	3.32	0.46	0.30^*^	0.46^*^	0.34^*^	0.43^*^			
6. Stewardship	3.21	0.36	0.16	0.40^*^	0.40^*^	0.69^*^	0.37^*^		
7. Humility	3.16	0.37	0.25^*^	0.39^*^	0.42^*^	0.81^*^	0.54^*^	0.60^*^	
8. Team performance	7.47	1.74	0.12	0.50^*^	0.44^*^	0.16	0.16	0.11	0.18

*n = 72*.

* *p < 0.05*.

### Conclusion

The results of the second study confirmed the findings of the first study, both in terms of the effect of shared servant leadership on team behavioral integration and the mediating role of information exchange in explaining the relationship between shared servant leadership and team performance. In addition, we were able to confirm once again the validity of the short version of the servant leadership measure. We now give a more general discussion on these findings and some indications for future research.

## Discussion

The research reported in this paper was designed to study the specific role of shared servant leadership in self-managed teams. The fact that we were able to replicate results in two studies separated by 1 year gives us confidence in our main findings. The most important findings were: (1) shared servant leadership has a very significant impact on team behavioral integration (confirming our first hypothesis), (2) information exchange plays a prominent role as a mediating variable between shared servant leadership and team performance (partially confirming the second hypothesis), and (3) a short measure of shared servant leadership was introduced consisting of four key dimensions (empowerment, humility, stewardship and accountability) and 15 items which appears to be valid and reliable. The results demonstrating the influence of shared servant leadership on team behavioral integration are a clear contribution to the servant leadership field. In a time when collectivist forms of leadership and self-managed teams seem to be gaining relevance in organizational work, it is interesting to notice how shared leadership processes and in particular shared servant leadership can be determinant in increasing collective behavior, information exchange and shared decision making. This confirms the perspective that leadership needs to be seen as a mutual process of taking ownership and initiative and not only as a one-to-others power relationship. There are multiple paths to creating teams that function, and centralized leadership can surely be one of them, but our results seem to demonstrate that shared leadership can also be quite effective in that process. Further research will be needed to understand the specific conditions under which shared leadership or centralized leadership become more appropriate for generating team behavioral integration. At the same time, through this study we show that servant leadership, with its focus on others, might be a model particularly suited for shared leadership in teams. Further investigating the role of each of the specific servant leadership dimensions on team behavioral integration will be important, while confirming the theoretical relationships established in this paper between these two constructs.

An essential theoretical contribution consists on a better understanding of the role of behavioral integration as a mediating variable between shared leadership and performance. Firstly, as explained before, team behavioral integration, already an important aspect in top management team performance (Simsek et al., 2005; Lubatkin et al., 2006), was shown to be influenced by the extent that team members showed servant leadership behavior toward each other. Our second finding suggests that information exchange was the most relevant dimension for the performance of self-managed teams in both our studies, which supports the importance attributed to this construct for shared leadership (Yammarino et al., 2012). We were however somehow surprised to observe that neither collective behavior nor joint decision making acted as mediating variables, as advanced in our hypothesis. It is likely, however, that the context will affect the relative importance of the separate team behavioral integration dimensions. We suggest that the particular influence of information exchange on team performance in our studies, compared to collective behavior and joint decision making, might have to do with the knowledge-intensive nature and short time span of the simulations in both assignments. In other words, when work is mainly related to the production of knowledge in a short period of time, the ability to quickly tap into the team's existing knowledge though effective information exchange might be the main driver of performance. This would be true for both information quantity (i.e., number of ideas and solutions being offered) and quality (i.e., relevance and effectiveness of the solutions). It seems therefore that the affective social integration aspect of collective behavior, demonstrated through behaviors of mutual support and social interaction, might be less relevant when teams need to work over short periods of time under high pressure. In addition, one should also note that the operationalization of joint decision making by Simsek et al. (2005) emphasizes that the needs and perspectives of different members are considered. The consequence being that joint decision processes are therefore by definition more time consuming. The short nature of the team assignments in this study and the need to quickly iterate between decisions and the simulation results (as opposed to highly complex decisions with multiple stakeholders over longer periods, as in the case of top management teams), might therefore help explain why joint decision making does not seem to play a relevant role for these particular cases. As such, we would expect collective behavior (as a measure of social integration and affective bond among team members) and joint decision making (as a time intensive task oriented dimension) to take an increasingly important role on performance over longer and more complex multi-stakeholder projects or when time pressure is not so high. Such contexts would be closer to the cases of top management teams, upon which most team behavioral integration literature has been developed. Further studies comparing short team assignments to longer and more complex team projects might shed some more light on this differentiated functioning of team behavioral integration.

Finally, the third finding of this paper on the short measure of shared servant leadership is rather promising from both a theoretical and practical perspective. The servant leadership survey that was the base of the current measure had to be modified to meet psychometric criteria and, at the same time, be practical for a round-robin approach of measuring shared leadership. A four-dimensional shared servant leadership scale is introduced that is in line with earlier theorizing on servant leadership (Van Dierendonck and Nuijten, 2011). The version that came out of our theoretical arguments and was confirmed in the analyses across two studies encompasses four core dimensions of servant leadership, namely: empowerment, humility, accountability and stewardship. We were able to observe that the short scale still represented 81% of the variance of the full scale. With only 15 items, instead of 30, this shortened survey can more easily be incorporated into future research on shared servant leadership and be of particular utility when using a round-robin approach with many mutual items between team members.

When considering the limitations of our study, we acknowledge that a student sample following a business simulation as the basis of the team work might not be entirely indicative of an organizational or business context. Regardless, the simulation's rather realistic scenarios, extracted from real-life cases does create a close-to-real environment when it comes to the actual decision being made. In addition our simulation based sample has multiple advantages. It guarantees the high response rate in most teams needed to test our hypothesis, which is very hard to realize in field studies. Teams had exactly the same assignment, eliminating the influence of aspects related to differing assignment complexities. The simulation took place within a limited time-span, being the main activity of the participants, reducing the influence of other work related demands in the team functioning. Also, most team studies use supervisory ratings of performance. Here it is feedback provided by the simulation program itself, which gives it a more objective and consistent character. Finally, a major strength is that we were able to replicate the findings of one study in the second study 1 year later, under the same circumstances and with the same type of assignment (something that would be very hard to realize outside an educational environment). At any rate, while there is supporting evidence to the parallels that can be established between students and other populations in their behavior in achievement settings (e.g., Locke, 1986; Brown and Lord, 1999), as our results seem to show, team behavioral integration might work differently depending on the duration, pressure and complexity of projects. Further studies in organizational or business settings would definitely be welcome to confirm our findings and further elaborate on the workings of team behavioral integration.

In conclusion, in view of the increasing popularity of collectivistic forms of leadership and self-managed teams in particular, getting additional insights into the processes that influence their effectiveness is crucial. The findings of this study emphasize the important role of shared servant leadership on team behavioral integration and its potential effect on performance through information exchange, further supporting the idea that servant leadership might be particularly suitable for shared leadership. Moreover, we are able to confirm the specific relevance of the four dimensions of empowerment, humility, accountability and stewardship as the key fundamental aspects of shared servant leadership, as well as the validity of the corresponding short measure.

## Author contributions

The article is the result of a joint effort and co-authorship is fully shared. The leading role shifted from the initial stages of field research to the final writing and editing of the document, as follows: Design and implementation of the field research (DV with the support of MS). Analysis of results (MS and DV jointly). Writing and editing of article (MS with the support of DV).

### Conflict of interest statement

The authors declare that the research was conducted in the absence of any commercial or financial relationships that could be construed as a potential conflict of interest.
